# Mothers’ satisfaction with care during facility-based childbirth: a cross-sectional survey in southern Mozambique

**DOI:** 10.1186/s12884-019-2449-6

**Published:** 2019-08-19

**Authors:** Sibone Mocumbi, Ulf Högberg, Erik Lampa, Charfudin Sacoor, Anifa Valá, Anna Bergström, Peter von Dadelszen, Khátia Munguambe, Claudia Hanson, Esperança Sevene, the CLIP working group, Sibone Mocumbi, Ulf Högberg, Erik Lampa, Charfudin Sacoor, Anifa Valá, Anna Bergström, Peter von Dadelszen, Khátia Munguambe, Claudia Hansonm, Esperança Sevene

**Affiliations:** 1 Department of Obstetrics and Gynaecology, Faculty of Medicine, Universidade Eduardo Mondlane (UEM), Av. Salvador Allende 702, 1100 Maputo, Mozambique; 2 Department of Women’s and Children’s Health, Uppsala University, Akademiska sjukhuset, SE-75185 Uppsala, Sweden; 3 Uppsala Clinical Research Centre, Uppsala University, Dag Hammarskjölds väg 38, 751 85 Uppsala, Sweden; 4 Centro de Investigação em Saúde de Manhiça (CISM), Rua 12, Manhiça, Mozambique; 5 University College London, Institute for Global Health, Gower St, London, WC1E 6BT UK; 6 Department of Women and Children’s Health, School of Life Course Sciences, Faculty of Life Sciences and Medicine, King’s College London, 1 Lambeth Palace Road, London, SE1 7EU UK; 7 Department of Public Health, Faculty of Medicine, Universidade Eduardo Mondlane, Av. Salvador Allende 702 R/C, Maputo, Mozambique; 8 Department of Public Health Sciences, Karolinska Institutet, Tomtebodavagen 18A, Plan 4, Stockholm, Sweden; 9 Department of Disease Control, London School of Hygiene and Tropical Medicine, Keppel St, London, WC1E 7HT UK; 10 Department of Physiological Science, Clinical Pharmacology, Faculty of Medicine, Universidade Eduardo Mondlane, Av. Salvador Allende 702 R/C, Maputo, Mozambique

**Keywords:** Satisfaction with care, Experiences of care, Facility-based childbirth

## Abstract

**Background:**

Client satisfaction is an essential component of quality of care. Health system factors, processes of care as well as mothers’ characteristics influence the extent to which care meets the expectations of mothers and families. In our study, we specifically aimed to address the mothers’ experiences of, and satisfaction with, care during childbirth.

**Methods:**

A population-based cross-sectional study, using structured interviews with published sequences of questions assessing satisfaction, including 4358 mothers who gave birth during the 12 months before June 2016 to estimate satisfaction with childbirth care. Regression analysis was used to determine the predictors of client satisfaction.

**Results:**

Most mothers (92.5%) reported being satisfied with care during childbirth and would recommend that a family member to deliver at the same facility. Specifically, 94.7% were satisfied with the cleanliness of the facility, 92.0% reported being satisfied with the interaction with the healthcare providers, but only 49.8% felt satisfied with the assistance to feed their baby. Mothers who had negative experiences during the process of care, such as being abandoned when needing help, disrespect, humiliation, or physical abuse, reported low levels of satisfaction when compared to those who had not had such experiences (68.5% vs 93.5%). Additionally, they reported higher levels of dissatisfaction (20.1% vs 2.1%). Regression analysis revealed that mothers who gave birth in primary level facilities tended to be more satisfied than those who gave birth in hospitals, and having a companion increased, on average, the overall satisfaction score, with 0.06 in type II health centres (CI 0.03–0.10) and with 0.05 in type I health centres (CI − 0.02 – 0.13), compared to − 0.01(CI -0.08 – 0.07) in the hospitals, irrespective of age, education and socio-economic background.

**Conclusion:**

Childbirth at the primary level facilities contributes to the level of satisfaction. The provision of childbirth care should consider women’s preferences and needs, including having a companion of choice. We highlight the challenge in balancing safety of care versus satisfaction with care and in developing policies on the optimum configuration of childbirth care. Interventions to improve the interaction with providers and the provision of respectful care are recommended.

**Electronic supplementary material:**

The online version of this article (10.1186/s12884-019-2449-6) contains supplementary material, which is available to authorized users.

## Background

Improving quality of care is fundamental to achieving Universal Health Coverage by 2030 [1]. The Universal Health Coverage goal emphasizes that health care systems should not only be designed to reduce the inacceptable burden of maternal deaths, stillbirths, and neonatal deaths that prevail in low- and middle-income countries [2], but also to offer care which meets the needs of the women, and is equitable and affordable. Patient satisfaction is a key part of quality of care [3] and, accordingly, the multidimensional aspects of quality of care provision are increasingly highlighted, as indicated in the World Health Organization’s (WHO) quality of care framework [4], which builds on the landmark article written by Donabedian [5]. These aspects include the need to address several of the underlying reasons for high mortality and insufficient care, which account for the prevailing unsatisfactory outcomes despite increases in uptake of care [4]. This framework highlights, effective communication, respect, and dignity, as well as, emotional support [4, 6], reflecting the growing concern about disrespect and abuse during childbirth care. Statements and initiatives have been formulated to reduce incidences of unacceptable treatment of women during pregnancy and childbirth [7–10].

Satisfaction with care during childbirth is a complex phenomenon consisting of multiple dimensions of satisfaction, as patients may be satisfied with one aspect of care but not with another, and experiences may change across different care providers’ components [11, 12]. A recently published review summarised that the following factors determine satisfaction with care: i) accessibility, ii) good physical environment, iii) cleanliness, iv) availability of drugs, supplies and human resources, v) level of care, vi) privacy and confidentiality, vii) promptness and viii) adequate emotional support [13]. While interest in research around experience and satisfaction with care has gained momentum, measurement issues prevail. The occurrence of disrespect and abuse depends on the context, the way in which the assessments are done, and the operationalization of their constructs [14]. Also, patient-level factors may determine reported levels of satisfaction. Women typically value facilities that are closer to home and facilities offering supportive care [15, 16], while overcrowding reduces client satisfaction [17]. Still, many women bypass primary facilities on the promise of receiving a better quality of care in higher-level facilities [18, 19]. In view of the policy shift towards recommending childbirth in higher- level and better-equipped facilities [20], it is important to increase the evidence base for the circumstances in which this care is provided and to develop an improved understanding of whether such centralised care responds sufficiently to the needs and expectations of mothers and families. In response, the objective of this study was to address the mothers’ experiences of, and satisfaction with, care during childbirth in a setting with a high facility-based childbirth rate.

## Methods

### Study design and participants

Using a cross-sectional household survey design, we interviewed women who recently gave birth (mothers), defined as those who gave birth during the 12 months prior to June 1st, 2016. These women were identified within a cohort established for the Community Level Interventions for Pre-eclampsia (CLIP) trial (NCT01911494) [21–23].

### Study setting

The study setting covered 12 selected rural areas, including 57,000 households within six districts of Maputo and Gaza provinces in southern Mozambique [22], with an institutional births coverage of 88.3 and 70.7%, respectively [24]. The population is largely rural and poor, and most derive a living from subsistence farming. Each area was purposively selected to reflect a variety of socioeconomic and demographic characteristics, such as level of urbanization, population density, and presence of a health facility. The study area includes 38 health facilities: 32 health centres (primary level facilities), and six hospitals (five secondary level and one tertiary level facilities). The health centres are classified as type I and type II. The type II health centres are the smallest ones, staffed by at least three nurses/midwives and offering outpatient services including reproductive and child health services and uncomplicated deliveries. The type I health centres are larger and have a more qualified team which includes a medical officer, and at least six nurses/midwives. In addition, they are equipped with basic laboratory and radiology sections. The hospitals are able to manage complicated deliveries, including performing caesarean section (C-section). More detailed information regarding the socioeconomic and demographic characteristics of the study setting have been described elsewhere [22].

### Theoretical framework and research instrument

**Fig. 1:**
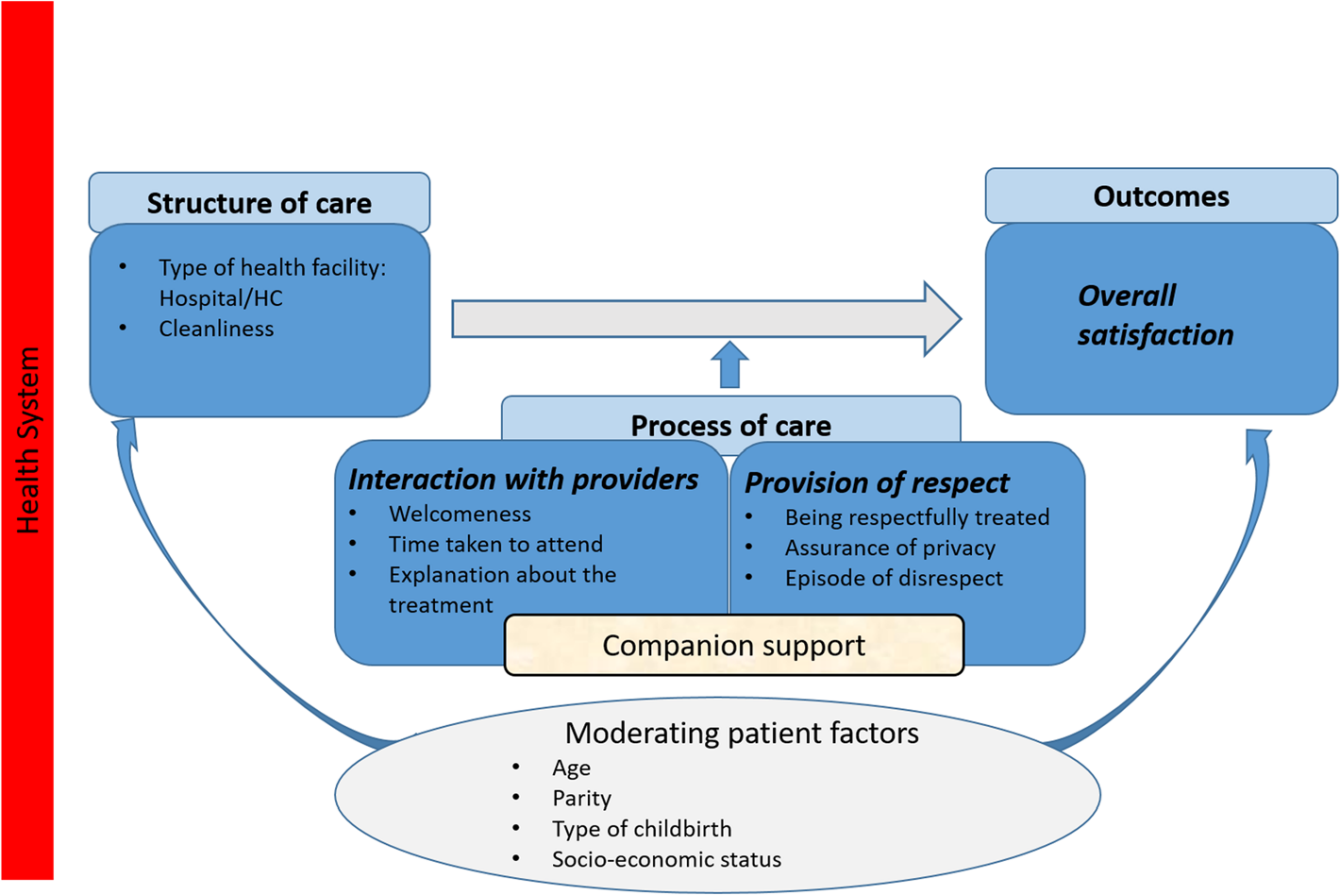
Conceptual framework of satisfaction with care during childbirth. Legend: The WHO’s quality of care framework for maternal and newborn health and the Srivastava’s frameworks have been utilized in defining the satisfaction items across each of the three Donabedian’s dimensions of quality of care: cleanliness of the facility and availability of medicines in the **structure of care** dimension, interaction with providers and the provision of respect in the **process of care** dimension, and the overall satisfaction of the mothers with the care provided during childbirth in the **outcome** dimension. Based on this framework, satisfaction is influenced by a variety of factors, and the mother’s level of satisfaction will depend on her evaluation of the distinct dimensions of childbirth. We treated companion support as a key effect modifier, and adjusted for other confounders. We defined, for the analysis, three dimensions: ***overall satisfaction***, ***interaction with providers*** and ***provision of respect***. The items included in the *overall satisfaction* dimension were those about the mothers’ satisfaction with childbirth care, and the level of recommendation for a relative to deliver in the same health facility. The *interaction with provider* dimension included items about the welcomeness and the willingness of the providers to attend, and about the clarity of the explanations provided. The *provision of respect* dimension comprised items about the experience of being respectfully treated, the way in which privacy was respected and the issue of disrespect

We developed a framework and satisfaction scores, which was adapted from the WHO’s quality of care framework for maternal and newborn health [4], and Srivastava’s conceptual framework of maternal satisfaction [13]. The framework conceptualizes satisfaction with care during childbirth by identifying three dimensions (derived from the Donabedian model of quality of care [5]) which should be addressed to assess the determinants of satisfaction: structure of care, the process of care, and outcomes (Fig. 1).

As our main aim was to address mothers’ experiences and their satisfaction with care provided during childbirth, we included in our outcome measure, the satisfaction score, and elements of structure, processes, and facility-based outputs.

Mothers’ satisfaction with care during childbirth was assessed using a questionnaire building on published sequences previously used to measure satisfaction [25, 26] (see Additional file 1). The first part of our questionnaire consisted of demographic information (age, parity, education, occupation, marital status, place of residence, religion, and household wealth). The second part consisted of questions relating to satisfaction, including elements of structure, process, and outcome, as well as events of disrespect and abuse. Structural elements included the type of health facility, cleanliness, and the availability of medicines. Process determinants included interaction with providers, provision of respect and privacy, and companion support [27, 28].

Overall satisfaction with the services was the main outcome. We used a 5-point Likert scale to measure mothers’ level of satisfaction (1-Very dissatisfied, 2-Dissatisfied, 3- Neutral 4-Satisfied, 5- Very satisfied). The questions were translated from English to Portuguese, and the questionnaire was pre-tested and piloted with a group of mothers of the Manhiça district, who were not members of the identified study population, to ensure that the questions were clear and understandable before application.

### Data collection

We programmed the questionnaire to be used on a tablet using ODK Collect version 1.4.6 [29, 30]. A total of 13 female data collectors and 12 field supervisors, trained for 2 weeks, visited and interviewed mothers at home between June 1st and October 28th, 2016. The training placed particular focus on the appropriate approaches to take when asking sensitive questions and on when to communicate the Portuguese questions in the local language (Changana). Attention was taken not to have anyone else but the mother present during the interviews. Data were uploaded and stored weekly to the Manhiça Health Research Centre (CISM). For data management and cleaning, the REDCap tool, version 6.14.0 (Vanderbilt University 2016) [31], was used. Data collectors were monitored by the field supervisors to ensure their compliance with the study protocol. The supervisors performed random second interviews with 1% of the mothers to test the quality of the data and to determine whether the data collectors needed re-training. Once a week, the principal investigator and the data management team reviewed both the completed questionnaire and the database to check for missing answers, duplications, and inconsistencies, and, if needed, the data collector was sent back to the field to gather data where corrections and clarifications were necessary.

### Outcome measures

The main outcome variable was the satisfaction of the mother with the care provided to her during childbirth, by answering the question “*Overall, taking everything into account, how are the services in the facility where you gave birth your last baby?*” and giving five response options. Also, we asked, “*If you now reconsider your birth experience, would you recommend a family member to deliver in the health facility where you gave birth*?”

We asked mothers about their perceptions about how welcoming the practitioners were at the health facility, the time taken to attend, clarity of the explanations, respectfulness while providing treatment, and respect for privacy during the physical examination. They were also asked about any experiences of disrespect, physical abuse, being abandoned when they needed help, and informal payment.

### Covariates

As independent variables, we included mothers’ socio-demographic characteristics (age, educational status, marital status, occupational status, religion and socio-economic status) and childbirth care characteristics: type of childbirth (vaginal/C-section), parity, district, health facility level, time spent in the health facility from admission till childbirth, presence of a companion during the childbirth and the fetal outcome (livebirth/stillbirth). Socio-economic status was estimated using principal components analysis on several household characteristics and assets.

### Statistical analysis

We used the statistic software R (version 3.4.3) for all analyses [32]. We recoded the answers on the Likert scale into three categories, which are, unsatisfied (very dissatisfied and dissatisfied), neutral, and satisfied (satisfied and very satisfied) and performed descriptive statistics to identify associations with socio-demographic determinants and childbirth characteristics using chi-square tests setting a 5% significance level.

Adapted from our framework, we defined for the analysis, three dimensions: *overall satisfaction*, *interaction with provider* and *provision of respect* (Fig. 1)*.* A confirmatory factor analysis was then fit to the data and scores for each of the dimensions were estimated for all individuals from the factor analysis model. The rationale behind the factor analysis was to reduce the total number of questions into a few dimensions that could be used for regression modelling. The comparative fit index for the confirmatory factor analysis was 0.98, and the root mean squared error of approximation was 0.08, indicating an acceptable fit to the data. Mothers who had been referred to a higher level health facility or had been admitted for C-section (and consequently had been attended at more than one facility) were excluded from the analysis, as it was not possible to identify to which health facility they were addressing their satisfaction level.

Robust linear models [33] were used to assess associations between the different dimensions of satisfaction and the independent variables. The robust linear model is similar to an ordinary least squares model but is less influenced by outliers [34, 35] and is 85% as efficient as the ordinary least squares model should the residual distribution be Gaussian.

A non-parametric bootstrap procedure was employed to account for the possible dependencies among individuals within each district. For each district, a bootstrap sample of the residuals was drawn. Each district was then designated a weight of + 1 or − 1 with a probability 0.5 each, and the district-specific bootstrap residuals were multiplied by that weight [36]. New pseudo-outcome values were then generated by adding the bootstrapped residuals to the fitted values from the original model and the analyses were re-run. A total of 5000 bootstrap replicates were made.

## Results

**Fig. 2:**
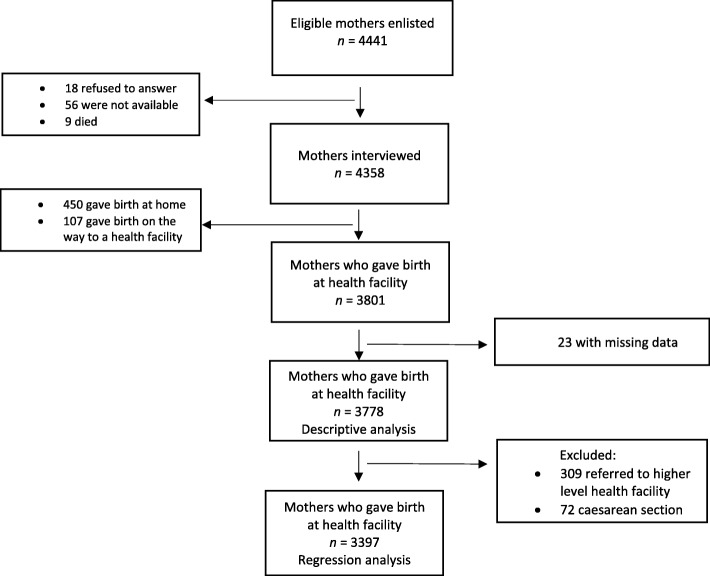
Diagram showing the flow through the study: mothers who gave birth within one year of the study start date in six districts of southern Mozambique, between 2015 and 2016

Overall, 4441 mothers were identified from the CLIP trial as having given birth during the 12 months before the start of the study, of which 83 (1.9%) were not interviewed because they were not found at home (65 mothers) or refused to answer (18 mothers), giving a response rate of 98.1%. Of the 4358 mothers interviewed, 3801 gave birth in a health facility (87.2%) and were asked about their satisfaction with the care during childbirth. We removed 23 respondents due to missing data, so 3778 mothers were considered for the descriptive analysis, but only 3397 were included in the regression analysis as 309 mothers who had been referred to a higher-level health facility or had been admitted for C-section (and consequently were attended at more than one facility) were excluded because it was not possible to identify to which health facility they were addressing their satisfaction level (Fig. 2).

### Socio-demographic characteristics of the mothers

The mothers’ median age was 25.0 years (range 14 to 49) and 60.6% were married. More than half (61.6%) had attended primary school, while 26.0% did not have any formal education. The majority were housewives (48.0%) or subsistence farmers (46.2%); only 2.8% were employed. Most (81.7%) were Christians. Half (52.6%) lived in households belonging to the poorest quintile groups (poor, poorer or poorest households) (Table 1).

**Table 1:** Sociodemographic characteristics of the 3778 mothers who gave birth in health facilities within 1 year of the study start date, in six districts of southern Mozambique between 2015 and 2016

Variables	Frequency	Percentage (%)
Median age (IQR) 25.0 (11.0) years		
Age in years by categories (range 14–49)		
≤ 19 years	828	21.9
20–34 years	2411	63.8
≥ 35 years	539	14.3
Completed educational level		
None	982	26.0
Primary	2327	61.6
Secondary or higher	469	12.4
Marital status		
Married	2289	60.6
Single	1398	37.0
Divorced/Widowed	91	2.4
Occupational status		
Housewife	1810	48.0
Subsistence farmer	1747	46.2
Student	114	3.0
Employed	107	2.8
Religion		
Zionist	1503	39.8
Other Protestants	1583	41.9
Catholics	507	13.4
Others	185	4.9
Socio-economic status		
Poorest	450	11.9
Poorer	681	18.0
Poor	856	22.7
Less poor	902	23.9
Least poor	733	19.4
Data missing	156	4.1

### Obstetric characteristics

The obstetric-related characteristics of the mothers are summarized in Table 2. Most (72.6%) were multiparas with a mean of 3.0 (SD ± 1.9) of previous births. More than half (53.4%) had a 1–5 km of distance to the nearest health facility. To reach the health facility, 50.3% walked and 29.8% used a taxi bus. More than one-third (43.2%) reported taking less than 30 min to travel from home to the health facility, while 39.6% took between 30 min and 1h. More than three quarters (76.5%) of the births occurred in primary level facilities and, of these, type II health centres were the most commonly used (78.5%). Spontaneous vaginal deliveries were the majority (95.8%); 0.5% had an assisted vaginal delivery and 3.7% a C-section - although it should be mentioned that the reported duration of labour was more than 24 h in 57.5% of the cases - and 2.3% mothers reported stillbirths. A companion was present in more than half of the deliveries (55.5%); the mother-in-law was the companion in 46.3% of these cases. Companionship during childbirth was mostly provided in the health centres (92.0% versus 8.0% in the hospitals).

**Table 2:** Mothers’ characteristics related to the day when they gave birth in health facilities, within one year of the study start date, in six districts of southern Mozambique between 2015 and 2016

Variables	Frequency	Percentage (%)
Parity (*n* = 3778)		
One	1037	27.4
Two to four	1937	51.3
Five and more	804	21.3
Districts where the mothers gave birth (*n* = 3778)		
Bilene-Macia	871	23.1
Chibuto	588	15.6
Chokwe	314	8.3
Magude	359	9.5
Manhiça	1109	29.3
Xai-Xai	537	14.2
Distance from home to the nearest health facility, km (*n* = 3753)		
< 1.0	619	16.5
≥ 1.0 to < 2.5	1108	29.5
≥ 2.5 to < 5.0	895	23.9
≥ 5.0 to < 7.5	433	11.5
≥ 7.5	698	18.6
Mean of transportation from home to the health facility (*n* = 3778)		
Walking	1900	50.3
Taxi mini bus	1127	29.8
Private car	557	14.7
Ambulance	161	4.3
Motorcycle/tuktuk/bicycle	33	0.9
Time took from home to the health facility (*n* = 3778)		
< 30 min	1633	43.2
≥ 30 min to < 1 h	1495	39.6
≥ 1 h to < 3 h	537	14.2
≥ 3 h	37	1.0
Don’t know	76	2.0
Type of health facility where the mothers gave birth (*n* = 3778)		
Type II health centre *	2268	60.0
Type I health centre **	621	16.4
Hospital	889	23.6
Referred to hospital (*n* = 3778)		
Yes	309	8.2
No	3469	91.8
Duration of labour (*n* = 3400)		
< 12 h	682	20.0
≥ 12 to < 24 h	764	22.5
≥ 24 h	1954	57.5
Type of childbirth (*n* = 3778)		
Spontaneous vaginally	3619	95.8
Ventouse	19	0.5
Caesarean section	140	3.7
Presence of companion throughout the childbirth (*n* = 3778)		
Yes	2095	55.5
No	1683	44.5
Type of companion (*n* = 2095)		
Mother-in-law	971	46.3
Mother	341	16.3
Husband/partner	106	5.1
Friend/neighbour	119	5.7
Others	558	26.6
Outcome (*n* = 3778)		
Livebirth	3691	97.7
Stillbirth	87	2.3

* Type II health centre – the smallest primary health care facilities, designed to serve between 7500 and 20,000 inhabitants, staffed by at least three nurses (one should be midwife) and one auxiliary nurse. They offer outpatient services including reproductive and child health services and uncomplicated deliveries

** Type I health centre – is bigger than type II and serves areas with between 16,000 and 35,000 inhabitants. The team is larger and more qualified. It includes a medical officer, at least six nurses (two should be midwives), one professional with basic qualifications for the pharmacy, one for the laboratory, and one for the radiology sections, and six auxiliary nurses

### Experiences of care and level of satisfaction with the services

Overall, 92.5% of the mothers were satisfied with services received, and 94.2% would recommend a family member to deliver in the same health facility (Table 3). Regarding structural facility indicators, 94.7% were satisfied with the cleanliness of the facility, and, among those who reported needing medicines during the childbirth, only 8.2% stated that they did not received them. Concerning process of care indicators, and specifically the interaction with the healthcare providers, most mothers (92.0% on average) reported being satisfied during labour; however, 10.0% reported having felt abandoned when they needed help. In contrast, only 49.8% of mothers felt satisfied with the assistance they received to feed their baby. Regarding the perceived levels of respect and privacy, most mothers (93.0% on average) were satisfied. However, disrespect or humiliation was reported by 6.3% of the mothers, being asked for informal payment by 4.2% and physical abuse by 1.7%. Among the 791 respondents who had any intervention (such as C-section or episiotomy), 40.3% reported the intervention was undertaken without being asked for consent.

**Table 3:** Satisfaction-related variables among mothers who delivered in health facilities within one year of the study start date in Maputo and Gaza provinces, Mozambique, between 2015 and 2016

Likert scores	Bad/No/Not satisfied	Neither good or bad/Undecided/ Neutral	Good/Yes/ Satisfied	
	*n* (%)	*n* (%)	*n* (%)	*N*
	[1,2]	[3]	[4,5]	
Overall satisfaction with the services				
Overall, taking everything into account, how are the services in the facility where you gave birth to your last baby?	116 (3.1%)	169 (4.5%)	3493 (92.5%)	3778
If you now reconsider your birth experience, would you recommend a family member to deliver in the health facility where you gave birth?	144 (3.8%)	77 (2.0%)	3557 (94.2%)	3778
Structure*				
How do you feel about the sanitation of the health facility? (How clean was it?)	76 (2.0%)	123 (3.3%)	3579 (94.7%)	3778
Process of care/Interaction with the healthcare providers**				
How did you feel about the way you were welcomed at this health facility?	88 (2.3%)	61 (1.6%)	3629 (96.1%)	3778
How do you feel about the time taken to attend to you during your delivery?	151 (4.0%)	147 (3.9%)	3480 (92.1%)	3778
How do you feel about the answers you received to your questions during your delivery?	31 (2.3%)	47 (3.4%)	1293 (94.3%)	1371
How would you rate the experience of how clearly the health providers explained things to you such as why something needed to be done?	114 (3.0%)	319 (8.4%)	3345 (88.5%)	3778
How would you rate the knowledge and competence of health workers at this facility for this delivery?	120 (3.2%)	120 (3.2%)	3538 (93.6%)	3778
How would you rate the experience of being helped by the health providers to feed your baby after your delivery?	907 (44.7%)	112 (5.5%)	1009 (49.8%)	2028
Provision of respect and privacy***				
During your delivery, how would you rate the experience of being respectfully treated?	94 (2.5%)	124 (3.3%)	3560 (94.2%)	3778
How would you rate the way privacy was respected during the physical examination?	125 (3.3%)	219 (5.8%)	3434 (90.9%)	3778
	**No**	**Yes**		
	***n*** **(%)**	***n*** **(%)**		***N***
Other questions related with the structure and process of care				
*If you needed special medicines during the delivery were you able to get the medicines the health provider prescribed?	40 (8.2%)	449 (91.8)%		489
**Did you feel abandoned when you needed help?	3387 (89.7%)	391 (10.3%)		3778
***Did the health providers ask you for consent before doing any intervention?	319 (40.3%)	472 (59.7%)		791
***Were you treated in a way that made you feel humiliated or disrespected?	3539 (93.7%)	239 (6.3%)		3778
***At any point during your stay for this delivery were you physically abused by any of the health care providers?	3715 (98.3%)	63 (1.7%)		3778

Mothers who had negative experiences during the process of care (such as disrespect or humiliation, physical abuse, being abandoned when they needed help, or being asked for informal payment) reported lower levels of satisfaction when compared to those who had not had such experiences: 68.2% vs 93.2% for disrespect or humiliation; 60.3% vs 93.0% for physical abuse; 67.8% vs 95.3% for being abandoned when they needed help, and 77.7% vs 93.1% for informal payment. In addition, they reported higher levels of dissatisfaction (reported as being “dissatisfied” or “very dissatisfied”): 20.5% vs 1.9% for disrespect or humiliation; 27.0% vs 2.7% for physical abuse; 23.0% vs 0.8% for being abandoned when they needed help, and 10.2% vs 2.8%.for informal payment (Table 4).

**Table 4:** Association of mothers’ negative experiences of care during childbirth with satisfaction outcome

Satisfaction outcome	Very dissatisfied		Dissatisfied		Neutral		Satisfied		Very satisfied			
	*n*	%	*n*	%	*n*	%	*n*	%	*n*	%	*N*	*p*
Experiences during childbirth												
Disrespect or humiliation											3778	< 0.001
Yes	20	8.4%	29	12.1%	27	11.3%	135	56.5%	28	11.7%	239	
No	20	0.6%	47	1.3%	142	4.0%	2078	58.7%	1252	35.4%	3539	
Physical abuse											3778	
Yes	7	11.1%	10	15.9%	8	12.7%	29	46.0%	9	14.3%	63	< 0.001
No	33	0.9%	66	1.8%	161	4.3%	2184	58.8%	1271	34.2%	3715	
Abandoned when needed help											3778	
Yes	30	7.7%	60	15.3%	36	9.2%	202	51.7%	63	16.1%	391	< 0.001
No	10	0.3%	16	0.5%	133	3.9%	2011	59.4%	1217	35.9%	3387	
Informal payment											3778	< 0.001
Yes	8	5.1%	8	5.1%	19	12.1%	72	45.9%	50	31.8%	157	
No	32	0.9%	68	1.9%	150	4.1%	2141	59.1%	1320	34.0%	3261	
Interventions undertaken without consent											3778	< 0.001
Yes	3	0.9%	10	3.1%	9	2.8%	101	31.7%	196	61.4%	319	
No	0	0.0%	0	0.0%	9	1.9%	95	20.1%	386	78.0%	472	
No intervention	37	1.2%	66	2.2%	151	5.1%	2017	67.5%	716	24.0%	2987	

### Association between satisfaction and mothers’ characteristics

In the bivariate analysis, satisfaction was positively associated with marital status (*p* < 0.001), being a subsistence farmer (*p* < 0.001), and those who were illiterate (*p* = 0.027). Additionally, living less than 2.5 km from the nearest health facility, having a means of transport to the health facility, having given birth in health centres, having less than 12h of labour duration and the presence of a companion (all *p* < 0.001), were also positively associated with mothers’ satisfaction (Table 5). A full table with all the mothers’ characteristics is provided as Additional file 2.

**Table 5:** Association of mothers’ characteristics with satisfaction outcome

Satisfaction outcome	Very dissatisfied		Dissatisfied		Neutral		Satisfied		Very satisfied			
	*n*	%	*n*	%	*n*	%	*n*	%	*n*	%	*N*	*p*
Sociodemographic characteristics												
Completed educational level											3778	0.027
None	3	0.6%	5	1.1%	16	3.4%	258	55.0%	187	39.9%	469	
Primary	31	1.3%	52	2.2%	101	4.3%	1364	58.6%	779	33.5%	2327	
Secondary or higher	6	0.6%	19	1.9%	52	5.3%	591	60.2%	314	32.0%	982	
Marital status											3778	< 0.001
Married	18	0.8%	42	1.8%	68	3.0%	1230	53.7%	931	40.7%	2289	
Single	22	1.6%	33	2.4%	96	6.9%	930	66.5%	317	22.7%	1398	
Divorced/Widowed	0	0.0%	1	1.1%	5	5.5%	53	58.2%	32	35.2%	91	
Occupational status											3778	< 0.001
Housewife	24	1.3%	36	2.0%	123	6.8%	1220	67.4%	407	22.5%	1810	
Subsistence farmer	16	0.9%	39	2.2%	37	2.1%	861	49.3%	794	45.4%	1747	
Student	0	0.0%	0	0.0%	5	4.4%	70	61.4%	39	34.2%	114	
Employed	0	0.0%	1	0.9%	4	3.7%	62	57.9%	40	45.4%	107	
Obstetric characteristics												
Distance to the nearest health facility in km											3753	< 0.001
< 1.0	5	0.8%	12	1.9%	26	4.2%	259	41.8%	317	51.2%	619	
≥ 1.0 to < 2.5	13	1.2%	16	1.4%	47	4.2%	541	48.8%	491	44.3%	1108	
≥ 2.5 to < 5.0	11	1.2%	18	2.0%	31	3.5%	580	64.8%	255	28.5%	895	
≥ 5.0 to < 7.5	2	0.5%	9	2.1%	35	8.1%	272	62.8%	115	26.6%	433	
≥ 7.5	9	1.3%	21	3.0%	29	4.2%	548	78.5%	91	13.0%	698	
Mean of transportation to the health facility											3778	< 0.001
Walking	30	1.6%	53	2.8%	79	4.2%	1111	58.5%	627	33.0%	1900	
Taxi mini bus	6	0.5%	17	1.5%	57	5.1%	636	56.4%	411	36.5%	1127	
Private car	3	0.5%	5	0.9%	25	4.5%	371	66.6%	153	27.5%	557	
Ambulance	0	0.0%	1	0.6%	6	3.7%	76	47.2%	78	48.4%	161	
Motorcycle/bicycle	1	3.0%	0	0.0%	2	6.1%	19	57.6%	11	33.3%	33	
Type of Health facility											3778	< 0.001
Type II health centre	29	1.3%	53	2.3%	90	4.0%	1268	55.9%	828	36.5%	2268	
Type I health centre	7	1.1%	19	3.1%	45	7.2%	359	57.8%	191	30.8%	621	
Hospital	4	0.4%	4	0.4%	34	3.8%	586	65.9%	261	29.4%	889	
Duration of labour											3400	< 0.001
< 12 h	12	1.8%	23	3.4%	15	2.2%	369	54.1%	263	38.6%	682	
≥ 12 to < 24 h	11	1.4%	25	3.3%	29	3.8%	469	61.4%	230	30.1%	764	
≥ 24 h	10	0.5%	24	1.2%	106	5.4%	1164	59.6%	650	33.3%	1954	
Presence of companion throughout the childbirth											3778	< 0.001
Yes	15	0.7%	35	1.7%	102	4.9%	1188	56.7%	755	36.0%	2095	
No	25	1.5%	41	2.4%	67	4.0%	1025	60.9%	525	31.2%	1683	

Regression analysis with robust linear models revealed that mothers who gave birth in health centres tended to be, overall, more satisfied than those who gave birth in the hospitals. Those who gave birth in type II health centres seemed to be the most satisfied after controlling for age, education and socio-economic index. Mothers who gave birth in health centres also tended to have higher satisfaction levels with their interaction with providers and the provision of respectful care, compared to those who gave birth in hospitals. Table 6 shows the estimated mean difference in the satisfaction dimension scores for having a companion versus not having a companion in health centres and hospitals. Comparing companionship, having a companion, increased, on average, the overall satisfaction score, with 0.06 in type II health centres (CI 0.03–0.10) and with 0.05 in type I health centres (CI -0.02 – 0.13). The same positive influence of companionship is observed on the scores for the interaction with providers and the provision of respectful care dimensions. The effect of companionship was lower in hospitals than in health centres for all the satisfaction dimension scores.

**Table 6:** Estimated mean difference in the satisfaction dimension scores for having a companion versus not having a companion in health centres (HC) and hospitals

	Estimate	Lower	Upper
Overall satisfaction			
Type I HC	0.05	-0.02	0.13
Type II HC	0.06	0.03	0.10
Hospital	−0.01	−0.08	0.07
Interaction with providers			
Type I HC	0.04	−0.04	0.11
Type II HC	0.06	0.02	0.09
Hospital	0.01	−0.07	0.08
Provision of respectful care			
Type I HC	0.07	−0.01	0.14
Type II HC	0.06	0.03	0.10
Hospital	−0.02	−0.09	0.05

## Discussion

### Main findings

Our findings drawn largely from mothers who experienced uncomplicated childbirth, indicate that most of them were satisfied with care during childbirth and would recommend a family member to deliver in the same facility. Mothers who gave birth in primary level facilities tended to be more satisfied than those who gave birth in hospitals, and the presence of a companion had a positive influence on the level of satisfaction, irrespective of age, education and socio-economic background. However, mothers who had negative experiences during childbirth, reported dissatisfaction more frequently than did those who had not had such experiences.

### Interpretation

High levels of satisfaction with care during childbirth have been reported in other low- and middle-income countries, such as in Ethiopia, Egypt, Malawi, Philippines, and Rwanda [37–43], and in high-income countries, such as in Australia, England and Sweden [44–46]. It has been questioned whether these high scores are an accurate representation of the mothers’ experiences [12]. Numerous explanations for high satisfaction scores have been discussed, such as met expectations being associated with lack of awareness regarding standards and client rights [47], lack of exposure to different care in a low literacy context [13], reluctance to express critical comments [48], timing and location of the interview [49], and different concept definitions [12, 13]. Alternatively, our findings could have reflected an unbiased perceived satisfaction, an interpretation supported by a linked qualitative study, in which we interviewed midwives, which revealed their commitment and devotion to the mothers despite resource constraints [50]. Conformity with social norms in relationships between patients and health providers could also play a role [51]. Contrary to our finding, Kifle et al. (2017) [52], reported a low rate of satisfaction associated with structural and process of care factors in Eritrea.

Despite the overall high satisfaction scores, 49.8% of mothers expressed their dissatisfaction with the support received for breastfeeding. It has been reported that dissatisfaction with care is easier to disclose when specific questions rather than global ones are asked [12]. As discussed by MacVicar et al. (2014), we cannot exclude the possibility that our findings reflect a type of support that is not culturally adequate and effective in meeting their breastfeeding intentions [53].

Results from Nepal indicated lower satisfaction among women delivering in overcrowded hospitals compared to smaller facilities [17], which also resonates well with our findings that satisfaction tended to be higher in primary level facilities. Tesfaye et al. (2016) [37] also reported lower satisfaction scores in hospitals compared to health centres. Considering the current debate on the safety of deliveries and the push for them to be conducted in fully equipped hospitals rather than in primary facilities [54], we believe that this finding is of great importance. In our study, proximity to a birthing facility and having a means of transport was important, and geographical accessibility has also been described in other studies as being significant for overall satisfaction [13]. This highlights the challenge inherent to balancing safety of care versus satisfaction of care, and the need to consider not only accessibility and proximity to home, but also service quality. Despite the finding of high satisfaction levels, some indicators found in our study are of concern in relation to the quality of care provided: the low rate (0.5%) of assisted vaginal delivery (AVD), the 3.7% C-section rate, and the high stillbirth rate (23 per 1000 births). Similar rates of less than 1% of institutional births delivered by AVD have been reported in several LMIC [55], for example, stillbirth rates as high as 25 per 1000 births [56]. The reasons most frequently described as contributing to the non-performance of AVD are a lack of trained human resources, lack of equipment, and national and institutional policies that fail to support AVD. The 3.7% C-section rate suggests unmet need [57], and this may be consistent with previous reports of underuse of C-section in rural areas of Mozambique [58].

Our findings have implications for policies on childbirth care in health care organisations. It is crucial to ensure that every woman delivers in a safe environment and that primary level facilities are enabled to provide evidence-based routine childbirth care and basic emergency obstetric care, as well as, referral capability for complicated cases.

Socio-demographic factors were of relatively minor importance to client satisfaction. Mehatu et al. (2017) were also unable to find an association between satisfaction and socio-demographic determinants in Nepal [17]. This finding is at odds with what has been published from studies in high-income countries, for example, Italy [59] and the Netherlands [60].

Relatively few mothers reported having experienced abuse and mistreatment. This finding might be rooted in the fact that the generation of women who deliver for the first time in facilities might perceive some forms of mistreatment, such as being shouted at, as normal [61, 62]. On the other hand, reports of levels of abuse and mistreatment depend on the method and timing used to assess it . Our interviewers might not have been sufficiently trained to ask probing questions to increase the number of reports of abuse provided by the women, and thus, our reported levels should be viewed with caution.

Client satisfaction with health care is subjective and is interlinked with expectations and outcomes of care [12]. While the concept of client satisfaction would profit from more stringent methodological development [63], the WHO framework of the quality of care for pregnant women and newborns puts forward three key dimensions of experience of care: i) effective communication, ii) respect and dignity, and iii) emotional support, which resonates well with what is consistently reported by mothers [13]. Beyond clinical arguments for continuous support for women during childbirth [27, 64], the fact that having a companion improved mothers’ satisfaction, underlines the importance of allowing a person of choice to accompany them during birth. Consistent with findings from other studies [16, 43, 65], interpersonal relationships were important contributors to patient satisfaction. Mothers who had negative experiences during the process of care reported dissatisfaction more frequently than did those who had not had such experiences. This aligns with previous reports on satisfaction with childbirth care [46]. Despite the low levels of dissatisfaction reported, further research is warranted to highlight more clearly the problems to be addressed for quality of care improvement [66].

### Strengths and limitations

The strength of our study is the population-based design, which minimizes ascertainment bias [22] and is likely to have minimized the risk of social desirability bias, which is suggested to reduce the reporting of dissatisfaction and mistreatment when assessments are done in facilities before discharge [67, 68]. Still, the data collectors, although not formally part of the health system, might have been perceived by the respondents as members of the health system (identified as members of CISM). Another strength is the size of the sample, which enhances the precision of the analysis, however missing data from 23 participants could have been a limitation.

The use of a cross-sectional study to assess satisfaction does not allow us to draw a conclusion about causality, but the research instrument explicitly referred to a previous event and confounding is unlikely. Recall bias might have been present, particularly as the interviews were conducted up to 1 year after birth. However, in contrast to the typical understanding of recall deteriorating over time, women’s self-reports of negative events, such as disrespect and abuse, may be more accurate when solicited after they have had some time to process their experiences, and in a setting away from the facility where they received maternity care.

The instrument we used to measure mothers’ satisfaction was adapted from previously validated instruments, and, despite being pre-tested and piloted, the risk of measurement bias cannot be excluded [69]. We addressed potential information bias resulting from the translation of questionnaires from Portuguese to local language by in-depth training of the interviewers. While our estimates must be interpreted with caution, we maintain that our analysis of drivers of satisfaction identifies essential aspects to be considered in further shaping the development of standardised measurements.

## Conclusion

Satisfaction with childbirth was driven by the proposed factors of communication, respect and dignity, and emotional support, as well as health systems factors. The majority of mothers were satisfied with the care they received during childbirth. The level of satisfaction was higher in women assisted in primary level facilities. Decisions on the configuration of childbirth care should ensure that every woman receives timely and evidence-based care and that providers consider women’s preferences and needs, including being able to have a companion of choice during childbirth. Interventions to improve interaction with providers and provision of respectful care are recommended.

## Additional files

Additional file 1: Study questionnaire. (PDF 1303 kb)

Additional file 2: Association of mothers’ characteristics with satisfaction outcome. (DOCX 45 kb)

## Data Availability

The datasets used and analysed during this study will be stored at the CISM repository and are available by request to the corresponding author after adhering to the CISM policy on data sharing.

## Abbreviations

CISM: Manhiça Health Research Centre
CLIP: Community Level Interventions for Pre-eclampsia
WHO: World Health Organization
