# Supplementary material for: Mothers’ satisfaction with care during facility-based childbirth: a cross-sectional survey in southern Mozambique
Source: BMC Pregnancy Childbirth. 2019 Aug 19;19:303. doi: 10.1186/s12884-019-2449-6 (PMC6701029; doi:10.1186/s12884-019-2449-6)
Supplement: Supplementary file 1 — Study questionnaire. (PDF 1303 kb) [file 12884_2019_2449_MOESM1_ESM.pdf]

Health survey with women having delivered during the last year

Instructions for interviewers about the questionnaire

The questions in this questionnaire have been set for the purpose of asking women about their own health and childbirth. First of all introduce yourself to the head of the household and to the women to be interviewed.

**Section A – Information about the interviewed woman**		
A1	Province	|__|__|
A2	District	|__|__|__|__|__|__|__|__|__|__|__|__|__| |__|__|__|__|__|__|__|__|__|__|__|__|__|
A3	Administrative post	|__|__|__|__|__|__|__|__|__|__|__|__|__| |__|__|__|__|__|__|__|__|__|__|__|__|__|
A4	Locality	|__|__|__|__|__|__|__|__|__|__|__|__|__| |__|__|__|__|__|__|__|__|__|__|__|__|__|
A5	Name of the village or burgh	|__|__|__|__|__|__|__|__|__|__|__|__|__| |__|__|__|__|__|__|__|__|__|__|__|__|__|
A6	Household ID	|__|__|- |__|__|__|__|-|__|__|__|
A6.1	Woman Perm_ID	|__|__|- |__|__|__|__|-|__|__|__|-|__|__|
A7	Interviewer code	|__|__|__|
A8	Date of today *(DD/MM/YYYY)*	|__|__|/|__|__|/|__|__|__|__|
A9	Is the identified mother present?<br>*If no, explain the reason of her absence and STOP the interview*<br>*For the women who delivered within the last one year, there are questions about the health of their pregnancy and childbirth. If the woman is not around, ask for the time that she will return, so that you come back again.* ***Try twice more.***	☐ Yes ☐ No
A10	*Read the informed consent form to the woman to be interviewed*<br>Do you agree to participate?<br>If no, STOP the interview	☐ Yes ☐ No
A11	Did the mother signed the informed consent?<br>If no, STOP the interview	☐ Yes ☐ No
A12	Starting time (*hour /minute*)	|__|__|:|__|__|
A13	Name of the woman	|__|__|__|__|__|__|__|__|__|__|__|__|__| |__|__|__|__|__|__|__|__|__|__|__|__|__|
A14	How old are you?	|__|__|
A16	Have you ever been pregnant?<br>*If not, STOP the interview*	☐ Yes ☐ No
A17	Have you ever been pregnant in the last 12 months?<br>*If not, STOP the interview*	☐ Yes ☐ No

A18	Last child born *(DD/MM/YYYY)*	|__|__|/|__|__|/|__|__|__|__|
A19	What is your marital status?<br><br>If the answer is " Married/union", please do answer A19.1	☐ Single<br>☐ Married/union<br>☐ Divorced<br>☐ Separated<br>☐ Widow<br>☐ Other: ___________________
A19.1	Type of union?	☐ Monogamous<br>☐ Poligamous
A20	Until which class did you study?<br>*If university degree put 13*	|__|__|
A20.1	If you studied in the university please indicate the degree completed	☐ Licentiate<br>☐ Master's degree<br>☐ PhD
A21	What is your main occupation?	☐ Housewife<br>☐ House maid<br>☐ Agriculture<br>☐ Small scale business<br>☐ Employed/technician/manager<br>☐ Student<br>☐ Other: ___________________
A22	Do you have other income generating activities?	☐ Yes ☐ No
A23	To which ethnic group to you belong?	☐ Changana<br>☐ Ronga<br>☐ Chope<br>☐ Chitswa<br>☐ Other: ______________________
A24	To which religion do you belong?	☐ Catholic<br>☐ Zionist<br>☐ Jehovah's witness<br>☐ Other Protestant<br>☐ Muslim<br>☐ Hindu<br>☐ Animist/traditional<br>☐ None<br>☐ Other: ___________________

**Section B - Module birth history**<br>*I would now like to ask you about your experience of pregnancy and childbirth. Some questions are about your experience in the health facility where you went. Please remember that nothing you tell us will be shared with the health facility and your responses will not negatively affect health care for you or your children in the future, There is no right or wrong answers to these questions. What is important is YOUR opinion. What we want to understand is YOUR experience.*		
B1	Did any of your pregnancies result into a miscarriage?<br>*Consider abortion as baby loss during the first 6 months of pregnancy, i.e. up to 27 weeks*<br>*If No go to B3*	☐ Yes ☐ No
B2	If yes, How many miscarriages did you have?	|__|__|
B3	How many children did you give birth to?	|__|__|
B4	So, in total, how many pregnancies did you have?	|__|__|
B5	Have you ever delivered a baby by cesarean section?	☐ Yes ☐ No
B6	If yes, how many cesarean sections did you have?	|__|__|
B7	Have you ever delivered a baby which was born dead?<br>*If no go to B9*	☐ Yes ☐ No
B8	If so, how many	|__|__|
B9	Have you ever delivered a child who had cried or showed some signs of being alive, but unfortunately died immediately afterwards?<br>*If no go to B11*	☐ Yes ☐ No
B10	If so, how many	|__|__|
B11	Did any of you babies born alive died later?<br>*If no go to B13*	☐ Yes ☐ No
B12	If so, how many	|__|__|
B13	Let me check again, it is right that you had |__|__| pregnancies<br>*If Yes go to B15*	☐ Yes ☐ No
B14	If B13 is not correct please clarify the number of pregnancies here.<br>*(Please note that if you make correction to this question, return to the previous one and correct where it was not right)*	|__|__|
Now, I am going to ask questions about your last delivery		
B15	Where did you deliver your last baby?<br>*If Health facility go to B16*	☐ Health facility<br>☐ On the way<br>☐ Home<br>☐ Other ________________________

B15.1	In the case you delivered outside of health facility, did you immediately go to health center/hospital after the delivery?<br>*If "* No*" go to B19*	☐ Yes ☐ No
B16	Please say the name of health post/health center/hospital where you delivered or had assistance after having delivered (chose from listed facilities below)	

		Distrito de BILENE- MACIA			Distrito de CHIBUTO
B16.1	☐	Centro de Saúde de Chissano	B16.21	☐	Centro de Saúde de Coca-Missava
B16.2	☐	Centro de Saúde de Licilo	B16.22	☐	Centro de Saúde de Malehice
B16.3	☐	Centro de Saúde da Macia	B16.23	☐	Centro de Saúde de Muavaquene
B16.4	☐	Centro de Saúde Mamonho	B16.24	☐	Centro de Saúde de Mucotuene
B16.5	☐	Centro de Saúde de Mazivila	B16.25	☐	Centro de Saúde de Txaimite
B16.6	☐	Centro de Saúde de Messano	B16.26	☐	Hospital Rural de Chibuto
B16.7	☐	Centro de Saúde de Olombe			**Distrito de MAGUDE**
		**Distrito de CHÓKWÉ**	B16.27	☐	Centro de Saúde Chicutso
B16.8	☐	Centro de Saúde de Hokwé	B16.28	☐	Centro de saúde de Magude
B16.9	☐	Centro de Saúde de Xilembene	B16.29	☐	Centro de Saúde de Mahele
B16.10	☐	Hospital Rural de Chókwé	B16.30	☐	Centro de Saúde de Moine
		**Distrito da MANHIÇA**	B16.31	☐	Centro de Saúde de Motaze
B16.11	☐	Centro de Saúde da Maragra	B16.32	☐	Centro de Saúde de Panjane
B16.12	☐	Centro de Saúde de Munguine			**Distrito de XAI-XAI**
B16.13	☐	Centro de Saúde de Maluana	B16.33	☐	Centro de Saúde de Chonguene
B16.14	☐	Centro de Saúde de Calanga	B16.34	☐	Centro de Saúde Siaia
B16.15	☐	Centro de Saúde de Malavele	B16.35	☐	Centro de Saúde Vlademir Lenine
B16.16	☐	Centro de Saúde de Palmeira	B16.36	☐	Hospital Provincial de Xai-Xai
B16.17	☐	Centro de Saúde 3 de Fevereiro	B16.37	☐	Hospital Rural de Chicumbane
B16.18	☐	Centro de Saúde de Taninga			**Outros**
B16.19	☐	Centro de Saúde Ilha Josina	B16.38	☐	Hospital Geral José Macamo
B16.20	☐	Hospital Distrital de Manhiça	B16.39	☐	Hospital Central de Maputo
B16.39	☐	Hospital Rural de Xinavane	B16.40	☐	Outros _________________

B17	Where you transferred during the delivery or after the delivery?<br>*If "* No*" go to B19*	☐ Yes ☐ No
B18	To what health center/hospital? (chose from list of health facility below)<br>(allow multiple answer in case the woman had subsequent referrals)	

		Distrito de BILENE- MACIA			Distrito de CHIBUTO
B18.1	☐	Centro de Saúde de Chissano	B18.21	☐	Centro de Saúde de Coca-Missava
B18.2	☐	Centro de Saúde de Licilo	B18.22	☐	Centro de Saúde de Malehice
B18.3	☐	Centro de Saúde da Macia	B18.23	☐	Centro de Saúde de Muavaquene
B18.4	☐	Centro de Saúde Mamonho	B18.24	☐	Centro de Saúde de Mucotuene
B18.5	☐	Centro de Saúde de Mazivila	B18.25	☐	Centro de Saúde de Txaimite
B18.6	☐	Centro de Saúde de Messano	B18.26	☐	Hospital Rural de Chibuto

	B18.7	☐	Centro de Saúde de Olombe		**Distrito de MAGUDE**		
		**Distrito de CHÓKWÉ**		B18.27	☐	Centro de Saúde Chicutso	
	B18.8	☐	Centro de Saúde de Hokwé	B18.28	☐	Centro de saúde de Magude	
	B18.9	☐	Centro de Saúde de Xilembene	B18.29	☐	Centro de Saúde de Mahele	
	B18.10	☐	Hospital Rural de Chókwé	B18.30	☐	Centro de Saúde de Moine	
		**Distrito da MANHIÇA**		B18.31	☐	Centro de Saúde de Motaze	
	B18.11	☐	Centro de Saúde da Maragra	B18.32	☐	Centro de Saúde de Panjane	
	B18.12	☐	Centro de Saúde de Munguine		**Distrito de XAI-XAI**		
	B18.13	☐	Centro de Saúde de Maluana	B18.33	☐	Centro de Saúde de Chonguene	
	B18.14	☐	Centro de Saúde de Calanga	B18.34	☐	Centro de Saúde Siaia	
	B18.15	☐	Centro de Saúde de Malavele	B18.35	☐	Centro de Saúde Vlademir Lenine	
	B18.16	☐	Centro de Saúde de Palmeira	B18.36	☐	Hospital Provincial de Xai-Xai	
	B18.17	☐	Centro de Saúde 3 de Fevereiro	B18.37	☐	Hospital Rural de Chicumbane	
	B18.18	☐	Centro de Saúde de Taninga		**Outros**		
	B18.19	☐	Centro de Saúde Ilha Josina	B18.38	☐	Hospital Geral José Macamo	
	B16.20	☐	Hospital Distrital de Manhiça	B18.39	☐	Hospital Central de Maputo	
	B16.39	☐	Hospital Rural de Xinavane	B18.40	☐	Outro ___________________	

I would like you to remember what happened on that time since the first symptoms of labour. I am going to use a timetable to help you to remember.

B19	At what time did you realize that labor started?	_____h (round to full hours)<br>☐ Don't know
B20	How long did it take you to make the decision to go to the facility after labor started or you had a problem?	_____h (round to full hours)<br>☐ N/A because went to health facility BEFORE labour started. *Go to B 26*<br><br>☐ N/A because didn't go to health facility. *Go to B 27*
B21	Who took the decision to go to the health facility?	☐ Herself<br>☐ Mother<br>☐ Mother in law<br>☐ Husband/partner<br>☐ Other relative<br>☐ Other: ____________________
B22	How long did it take you to go out home, after having decided to go to the health facility?	_____h (round to full hours)
B23	How long did it take you to travel to the facility?	_____h (round to full hours)
B24	How long did they take to attend you at the health facility?	_____h (round to full hours)
B25	Did you receive any medication in the facility to speed up labour?	☐ Yes ☐ No
B26	Did you take any herbal medicine to speed up labor?	☐ Yes ☐ No
B27	When the baby was born?	_____h (round to full hours)
B28	[automatic calculation]<br>*How long did the labor take in total, from the first moment the mother realized that birth started to the time the baby was born?*	____day ___ hours (round to full hours)

B29	Was the baby delivered alone or with its twin?<br>*If single go to B31*	☐ Single<br>☐ Multiple
B30	If it were twins, how many?	|__|__|
B31	How did you deliver your last baby?<br>*Give women the choices and probe for ventouse and breech delivery. Explain what is cesarean section, ask if her abdomen was open to deliver the baby*<br>[answer for each twin if it is the case]<br>*(skip to B35 if NOT a Caesarean section)*	☐ Spontaneous vertex (vaginally)<br>☐ Breech vaginally<br>☐ Ventouse<br>☐ Caesarian section
B32	Have you had problem with wound dehiscence?<br>*Did the wound open?*	☐ Yes ☐ No
B33	Have you had problem with infection in the scar?<br>*Probe…fever, antibiotic*	☐ Yes ☐ No
B34	Have you or have you had pain in the scar?	☐ Yes ☐ No
B35	Who supported you when delivering this child?<br>Tick ALL that applies<br><br>*Probe the type of the person who supported her, and list down each person*.	☐ Doctor<br>☐ Clinical officer (Técnico de cirurgia)<br>☐ Nurse/Midwife<br>☐ Auxiliary Nurse/midwife<br>☐ Traditional birth attendant<br>☐ Relative<br>☐ Other: ____________________<br>☐ None<br>☐ Don't know
B36	Do you have a Caderneta de Saúde da Mulher, do you allow me to take some information from this care?	☐ Yes ☐ No
B37	Was the height of the mother recorded as being below 150 cm?	☐ Yes ☐ No ☐ Missing
B38	Could you check what the birth weight of the baby was? (info from *Caderneta de Saúde da Mulhe*r)<br>[answer for each twin if it is the case]	|_|_|_|_| grams ☐ Not available from the caderneta
B38.1	If not available from the caderneta, do you know what the birth weight of the baby was?<br>[answer for each twin if it is the case]	☐ Yes |_|_|_|_| gram<br>☐ No
B39	Could you check what the head circumference of the baby was? (info from *Caderneta de Saúde da Mulhe*r Recem nascido Perimetro cranenano)<br>[answer for each twin if it is the case]	|__|__| cm ☐ Missing
B40.1	Could you check what the 1st minute Apgar score of the baby was? (info from *Caderneta de Saúde da Mulhe*r Recem nascido Recem nascido Apgar (Check *Caderneta de Saúde da Mulhe*r)<br>[answer for each twin if it is the case]	APGAR |__| 1 min<br><br>☐ Missing
B40.2	Could you check what the 5th minute Apgar score of the baby was? (info from *Caderneta de Saúde da Mulhe*r Recem nascido Recem nascido Apgar (Check *Caderneta de Saúde da Mulhe*r)<br>[answer for each twin if it is the case]	APGAR |__| 5 min<br><br>☐ Missing

B41	Have you been tested for HIV?	☐ Yes ☐ No ☐ Missing
B42	What is the result (Check *Caderneta de Saúde da Mulher*)	☐ Positive ☐ Negative<br>☐ Undetermined
B43	Have you been submitted to surgery up to 42 days after delivery?<br>*if No jump to Section C Fistula module*	☐ Yes ☐ No
B44	Does your uterus has been removed?<br>*If Yes jump to Section C Fistula module*	☐ Yes ☐ No ☐ Don't know
B45	If the uterus has not been removed in that surgery, do you know what kind of procedure has been done?<br>*Check if you have any hospital document to complete the information provided (hospital card, letter of hospital discharge, referral letter, other…)*	☐ Yes ☐ No<br><br>If Yes, describe the procedure:<br>___________________________

Section C - Fistula module

Sometimes a woman can have a problem such that she experiences a constant leakage of urine or stool from her vagina during the day or night. It also can happen that a women has this problem only from time to time when coughed, laughed, sneezed etc.

C1	Do you know that constant leakage of urine or stool from your vagina can be a disastrous complication after birth?	☐ Yes ☐ No
C2	Have you heard about women's rights to get proper treatment in case of suffering from constant leakage of urine or stool from your vagina?	☐ Yes ☐ No
C3	Have you, BEFORE your last pregnancy, ever experienced a constant leakage of urine or stool from your vagina during the day and night?	☐ Yes ☐ No ☐ Don't know
C4	Have you AFTER your last pregnancy ever experienced a constant leakage of urine or stool from your vagina during the day and night?<br><br>*If No, go to Section D - Perceived health and pain*	☐ Yes ☐ No ☐ Don't know
C5	How would you rate your last labor? Was it normal, or difficult or even very difficult?	☐ Normal<br>☐ Difficult<br>☐ Very difficult
C6	What kind of leakage do you experience?	☐ Urine only<br>☐ Urine and stool<br>☐ Stool only
C7	How many days after delivery did the leakage start?	|__|__|

C8	In the past 7 days, have you lost or leaked urine when you coughed, laughed, sneezed etc.?	☐ Yes ☐ No ☐ Don’t know
C9	Has it been a big, little or no problem for you	☐ Big problem<br>☐ Little problem<br>☐ No problem
C10	Have you sought treatment for this condition?<br><br>*If Yes go to C12*	☐ Yes ☐ No
C11	If No, Why have you not sought treatment?<br>*Tick ALL that applies*<br><br>*Go to module perceived health and pain after answering this question*	☐ Did not know problem could be fixed<br>☐ Do not know where to go<br>☐ Too expensive<br>☐ Too far to reach treatment facility<br>☐ Poor quality of care at treatment facility<br>☐ Could not get permission to go<br>☐ Embarrassment<br>☐ Other: ___________________
C12	If yes, where did you seek treatment?	☐ Health center<br>☐ Hospital<br>☐ Other: ___________________
C13	Have you been operated?	☐ Yes ☐ No
C14	From whom did you last seek treatment?	☐ Doctor/<br>☐ Clinical officer (Técnico de Cirurgia)<br>☐ Nurse/Midwife<br>☐ Auxiliary nurse<br>☐ Traditional healer<br>☐ Traditional birth attendant<br>☐ APE<br>☐ Religious activist<br>☐ Other: ___________________

Section D - Perceived health and pain

I would now like to ask you about how you perceive your health now.

For this section you might use the smiley

D1	How do you perceive your overall health now?	☐ Very good<br>☐ Good<br>☐ Neither good or bad<br>☐ Bad<br>☐ Very bad
D2	Have you had any pain within the last 24 hours?<br><br>*If No go to D7*	☐ Yes ☐ No
D3	Where do you feel pain or have felt pain in the past 24 hours.<br><br>*Tick ALL that applies*	☐ In your back<br>☐ In your head<br>☐ On the *outside* of your abdomen or front<br>☐ *Deep inside* your abdomen or front<br>☐ In your bottom or genital area<br>☐ Other: ___________________

D4	How severe is the *worst* pain you have had in the last 24 hours?<br><br>*Mark ONLY the answer that best describes the pain the woman explains*	☐ Almost no pain<br>☐ Mild or a small amount of pain<br>☐ Quite a lot of pain<br>☐ Severe or excruciating<br>☐ Terrible pain
D5	Have you taken any kind of tablets or medicine for the pain in the last 24 hours?	☐ Yes ☐ No
D6	Have you taken any kind of traditional medicine for the pain in the last 24 hours?	☐ Yes ☐ No
D7	If you have had sex since the birth of your baby, was it painful on the most recent occasion?<br><br>*If No OR Have not had sex BUT delivered with caesarian section go to Section E*	☐ Yes ☐ No<br>☐ Have not had sex
D8	How severe was the pain?	☐ Almost no pain<br>☐ Mild or a small amount of pain<br>☐ Quite a lot of pain<br>☐ Severe or excruciating<br>☐ Terrible pain

Section E - Health system delivery service responsiveness module (including perceived quality and satisfaction, experience of disrespect…)

Now I would like to ask you a few questions on your last delivery, how you felt about the service provided to you and how satisfied you were with your birth experience.

Some questions I have, relate to how you feel about you were taken care of at the facility where you went. For those questions you can use this "ruler" [present the smiley options showing the different alternatives to the woman]. *I will let you know when this can be applied.*

E1	Why did you chose the place where you had your delivery? (tick all that apply)	☐ Nearest facility<br>☐ Best facility/clean environment/good services<br>☐ Same facility used for ANC<br>☐ To get services not available elsewhere<br>☐ Hospital of choice for delivery<br>☐ Affordable services<br>☐ Recommended by relative/friend<br>☐ Avoid congestion in other facilities<br>☐ Referred from another facility<br>☐ Well equipped facility with supplies<br>☐ Only option<br>☐ Facility offer specialized services<br>☐ Because of complications<br>☐ Providers treat clients well<br>☐ Decision made by spouse<br>☐ Like the facility<br>☐ Other: ____________________

E2	How did you get to the place where you delivered?<br><br>*If N/A or delivered at home, skip to E7*	☐ Ambulance<br>☐ Private car<br>☐ Taxi motorcycle/Tutuk<br>☐ Stretcher<br>☐ Bicycle<br>☐ Walked<br>☐ Other: ___________________<br>☐ N/A (delivered at home)
E3	Approximately how many hours did it take you to travel to the place where you had your delivery?	☐ 0 - 30 minutes<br>☐ up to 1 hour<br>☐ 1 – 3 hours<br>☐ more than 3 hours<br>☐ Don’t know<br>☐ N/A (delivered at home)
E4	Who accompanied you to the place where you gave birth?	☐ No one<br>☐ Mother<br>☐ Mother-in-law<br>☐ Husband/partner<br>☐ Other relative<br>☐ Other: ___________________<br>☐ N/A (delivered at home)
E5	How did you feel about the way you were welcomed at this health facility?	☐ Very unhappy ☺<br>☐ Unhappy ☺<br>☐ Neither unhappy nor happy 😐<br>☐ Happy ☹<br>☐ Very happy☹<br>☐ N/A (delivered at home)
E6	How do you feel about the sanitation of the health facility? (How clean was it?) Pictures….	☐ Very unhappy ☺<br>☐ Unhappy ☺<br>☐ Neither unhappy nor happy 😐<br>☐ Happy ☹<br>☐ Very happy☹
E7	Were you allowed to have someone accompanying you throughout your delivery?<br>*If no, go to E9*	☐ Yes ☐ No ☐ Don’t know
E8	If yes, who accompanied you throughout your delivery?	☐ Mother<br>☐ Mother-in-law<br>☐ Husband/partner<br>☐ Other relative<br>☐ Friend/neighbor<br>☐ Traditional birth attendant<br>☐ Primary health care agent (APE)<br>☐ Other: ___________________
E9	During your delivery, how would you rate the experience of being respectfully treated? I mean being treated with care and attention	☐ Very good<br>☐ Good<br>☐ Neither good or bad<br>☐ Bad<br>☐ Very bad

E10	Did you fell abandoned when you needed help? *For example, did you ask for help and nobody come?*	☐ Yes<br>☐ No, Go to ….E12
E11	When?<br>*(DO NOT READ, Circle all that apply, prompt for any more)*	☐ While in labor<br>☐ While delivering<br>☐ While experiencing a complication<br>☐ After delivery<br>☐ Baby after delivery<br>☐ Other _____________________
E12	How do you feel about the time taken to attend to you during your delivery?	☐ Very unhappy ☺<br>☐ Unhappy ☺<br>☐ Neither unhappy nor happy 😐<br>☐ Happy ☹<br>☐ Very happy☹
E13	On a scale of 1-5 were you treated in a way that made you feel humiliated or disrespected?<br>A 5 means *"I did not feel humiliated or disrespected"*. A 1 mean *"I feel very humiliated or disrespected"*.	☐ Very humiliated or disrespected 1<br>☐ Not very humiliated/disrespected 2<br>☐ Somewhat humiliated/disrespected 3<br>☐ A little humiliated/disrespected 4<br>☐ Not humiliated/disrespectfully treated5
E13B	Let me check again: did you deliver in a health facility? If you delivered at home or on the way, have you got assistance in a health unit after the delivery?	☐ Yes<br>☐ No, *STOP the interview*
**Question ONLY for women who delivered at HEALTH FACILITY**		
E14	At any point during your stay for this delivery were you physically abused by any of the health care workers? For example physical abuse might include being hit or slapped.	☐ Yes<br>☐ No, *GO to E17*
E15	What exactly happened? (*DO NOT READ, tick all that apply, prompt for any more*)	☐ Shouted<br>☐ Kicked<br>☐ Pinched<br>☐ Slapped<br>☐ Pushed<br>☐ Beaten<br>☐ Tied to the delivery bed/delivery coach<br>☐ Other _____________________
E16	On a scale of 1 to 5 how bad did you feel/how much did you suffer as a result of being physically disrespected?<br>*a 5 means "I did not feel bad or suffer at all." a 1means you felt the worst you could possibly feel/suffer*	☐ Felt very bad 1<br>☐ Not very bad 2<br>☐ Somewhat bad 3<br>☐ A little bad 4<br>☐ Not bad at all 5
E17	How would you rate the way privacy was respected during the physical examination?	☐ Very good<br>☐ Good<br>☐ Neither good or bad<br>☐ Bad<br>☐ Very bad

E18	How would you rate the experience of how clearly the health providers explained things to you such as why something needed to be done?	☐ Very good<br>☐ Good<br>☐ Neither good or bad<br>☐ Bad<br>☐ Very bad
E19	Did the health providers ask you for consent before doing any intervention?	☐ Yes ☐ No ☐ Don't know<br>☐ N/A (no intervention done)
E20	How do you feel about the answers you received to your questions during your delivery?	☐ Very unhappy ☺<br>☐ Unhappy ☺<br>☐ Neither unhappy nor happy 😐<br>☐ Happy ☹<br>☐ Very happy☹
E21	If you needed special medicines during the delivery were you able to get the medicines the health provider prescribed	☐ Yes, all were available *go to E23*<br>☐ Most were available<br>☐ Some were, some not<br>☐ Very few were available<br>☐ None of them was available<br>☐ Did not need special medicines *go to E23*
E22	Which reasons explain why you were not able to get the medicines you were prescribed	☐ Could not afford<br>☐ Were not available<br>☐ Did not believe that all the medicines were needed<br>☐ Started to feel better<br>☐ Already had some medicines at home<br>☐ Other: ___________________
E23	Did you pay officially to have access to the health unit for your delivery?	☐ Yes ☐ No
E24	At any point during this facility did you feel/perceive or were asked by anyone for money other than the official costs of the service to access services or any favor?	☐ Yes ☐ No *(go to question E26)*
E25	How much did you pay for the above?	_____________________ MT
E26	If you now reconsider your birth experience, would you recommend a family member to deliver in the health facility where you delivered?	☐ Yes, very much<br>☐ Yes<br>☐ Undecided<br>☐ No<br>☐ Not at all
E27	How would you rate the knowledge and competence of health workers at this facility for this delivery?	☐ Very good<br>☐ Good<br>☐ Neither good or bad<br>☐ Bad<br>☐ Very bad
E28	Overall, taking everything into account, how are the services in the facility where you delivered your last baby?	☐ Very good<br>☐ Good<br>☐ Neither good or bad<br>☐ Bad<br>☐ Very bad

**Specific question for women who received a Caesarean section**		
E29	What do you personally think: Do you feel that the Caesarean section was necessary?	☐ Yes ☐ No ☐ Don't know
**Question ONLY for women who had a LIVE birth**		
E30	How would you rate the experience of being helped by the health providers to feed your baby after your delivery?	☐ Very unhappy ☺<br>☐ Unhappy ☺<br>☐ Neither unhappy nor happy 😐<br>☐ Happy ☹<br>☐ Very happy ☹

Thank the women and end the interview

	Finishing time *hour /minute*	|__|__|:|__|__|
	Signature (interviewer code)	
