# Supplementary material for: Mothers’ satisfaction with care during facility-based childbirth: a cross-sectional survey in southern Mozambique
Source: BMC Pregnancy Childbirth. 2019 Aug 19;19:303. doi: 10.1186/s12884-019-2449-6 (PMC6701029; doi:10.1186/s12884-019-2449-6)
Supplement: Supplementary file 2 — Association of mothers’ characteristics with satisfaction outcome. (DOCX 45 kb) [file 12884_2019_2449_MOESM2_ESM.docx]

**Additional file 2: Table S1** Association of mothers’ characteristics with satisfaction outcome

|  | **Satisfaction outcome** | **Very dissatisfied** | | **Dissatisfied** | | **Neutral** | | **Satisfied** | | **Very satisfied** | |  |  |
| --- | --- | --- | --- | --- | --- | --- | --- | --- | --- | --- | --- | --- | --- |
|  |  | ***n*** | **%** | ***n*** | **%** | ***n*** | **%** | ***n*** | **%** | ***n*** | **%** | ***N*** | ***p*** |
| **Sociodemographic**  **characteristics** | | | | |  |  |  |  |  |  |  |  |  |
| Age by groups | |  |  |  |  |  |  |  |  |  |  | 3778 | 0.913 |
|  | ≤19 years | 8 | 1.0% | 14 | 1.7% | 39 | 4.7% | 504 | 60.9% | 263 | 31.8% | 828 |  |
|  | 20–34 years | 26 | 1.1% | 50 | 2.1% | 106 | 4.4% | 1401 | 58.1% | 828 | 34.3% | 2411 |  |
|  | ≥35 years | 6 | 1.1% | 12 | 2.2% | 24 | 4.5% | 308 | 57.1% | 189 | 35.1% | 539 |  |
| Completed educational level | |  |  |  |  |  |  |  |  |  |  | 3778 | 0.027 |
|  | None | 3 | 0.6% | 5 | 1.1% | 16 | 3.4% | 258 | 55.0% | 187 | 39.9% | 469 |  |
|  | Primary | 31 | 1.3% | 52 | 2.2% | 101 | 4.3% | 1364 | 58.6% | 779 | 33.5% | 2327 |  |
|  | Secondary or higher | 6 | 0.6% | 19 | 1.9% | 52 | 5.3% | 591 | 60.2% | 314 | 32.0% | 982 |  |
| Marital status | |  |  |  |  |  |  |  |  |  |  | 3778 | <0.001 |
|  | Married | 18 | 0.8% | 42 | 1.8% | 68 | 3.0% | 1230 | 53.7% | 931 | 40.7% | 2289 |  |
|  | Single | 22 | 1.6% | 33 | 2.4% | 96 | 6.9% | 930 | 66.5% | 317 | 22.7% | 1398 |  |
|  | Divorced/Widowed | 0 | 0.0% | 1 | 1.1% | 5 | 5.5% | 53 | 58.2% | 32 | 35.2% | 91 |  |
| Occupational status | |  |  |  |  |  |  |  |  |  |  | 3778 | <0.001 |
|  | Housewife | 24 | 1.3% | 36 | 2.0% | 123 | 6.8% | 1220 | 67.4% | 407 | 22.5% | 1810 |  |
|  | Subsistence farmer | 16 | 0.9% | 39 | 2.2% | 37 | 2.1% | 861 | 49.3% | 794 | 45.4% | 1747 |  |
|  | Student | 0 | 0.0% | 0 | 0.0% | 5 | 4.4% | 70 | 61.4% | 39 | 34.2% | 114 |  |
|  | Employed | 0 | 0.0% | 1 | 0.9% | 4 | 3.7% | 62 | 57.9% | 40 | 45.4% | 107 |  |
| Religion | |  |  |  |  |  |  |  |  |  |  | 3778 | <0.001 |
|  | Zionist | 13 | 0.9% | 23 | 1.5% | 62 | 4.1% | 892 | 59.3% | 513 | 34.1% | 1503 |  |
|  | Other Protestants | 17 | 1.1% | 34 | 2.1% | 71 | 4.5% | 871 | 55.0% | 590 | 37.3% | 1583 |  |
|  | Catholics | 8 | 1.6% | 14 | 2.8% | 24 | 4.7% | 335 | 66.1% | 126 | 24.9% | 507 |  |
|  | Others | 2 | 1.1% | 5 | 2.7% | 12 | 6.5% | 115 | 62.2% | 51 | 27.6% | 185 |  |
| Socio-economic status | |  |  |  |  |  |  |  |  |  |  | 3622 | 0.1297 |
|  | Poorest | 1 | 0.2% | 5 | 1.1% | 21 | 4.7% | 270 | 60.0% | 153 | 34.0% | 450 |  |
|  | Poorer | 10 | 1.5% | 16 | 2.3% | 32 | 4.7% | 402 | 59.0% | 221 | 32.5% | 681 |  |
|  | Poor | 6 | 0.7% | 18 | 2.1% | 31 | 3.6% | 514 | 60.0% | 287 | 33.5% | 856 |  |
|  | Less poor | 12 | 1.3% | 24 | 2.7% | 47 | 5.2% | 509 | 56.4% | 310 | 34.4% | 902 |  |
|  | Least poor | 9 | 1.2% | 11 | 1.5% | 28 | 3.8% | 431 | 58.8% | 254 | 34.7% | 733 |  |
| **Obstetric characteristics** | | |  |  |  |  |  |  |  |  |  |  |  |
| Parity | |  |  |  |  |  |  |  |  |  |  | 3778 | 0.8361 |
|  | One | 11 | 1.1% | 18 | 1.7% | 49 | 4.7% | 617 | 59.5% | 342 | 33.0% | 1037 |  |
|  | Two to four | 18 | 0.9% | 44 | 2.3% | 85 | 4.4% | 1139 | 58.8% | 651 | 33.6% | 1937 |  |
|  | Five and more | 11 | 1.4% | 14 | 1.7% | 35 | 4.4% | 457 | 56.8% | 287 | 35.7% | 804 |  |
|  |  |  |  |  |  |  |  |  |  |  |  |  |  |
| District where the mothers  gave birth | |  |  |  |  |  |  |  |  |  |  | 3778 | <0.001 |
|  | Chibuto | 10 | 1.7% | 28 | 4.8% | 14 | 2.4% | 484 | 82.3% | 52 | 8.8% | 588 |  |
|  | Bilene-Macia | 18 | 2.1% | 31 | 3.6% | 71 | 8.2% | 429 | 49.3% | 322 | 37.0% | 871 |  |
|  | Chokwe | 0 | 0.0% | 1 | 0.3% | 4 | 1.3% | 4 | 1.3% | 305 | 97.1% | 314 |  |
|  | Magude | 1 | 0.3% | 0 | 0.0% | 10 | 2.8% | 325 | 90.5% | 23 | 6.4% | 359 |  |
|  | Manhiça | 8 | 0.7% | 9 | 0.8% | 32 | 2.9% | 525 | 47.3% | 535 | 48.2% | 1109 |  |
|  | Xai-Xai | 3 | 0.6% | 7 | 1.3% | 38 | 7.1% | 446 | 83.1% | 43 | 8.0% | 537 |  |
| Distance to the nearest health facility in km | | | |  |  |  |  |  |  |  |  | 3753 | <0.001 |
|  | < 1.0 | 5 | 0.8% | 12 | 1.9% | 26 | 4.2% | 259 | 41.8% | 317 | 51.2% | 619 |  |
|  | ≥ 1.0 to <2.5 | 13 | 1.2% | 16 | 1.4% | 47 | 4.2% | 541 | 48.8% | 491 | 44.3% | 1108 |  |
|  | ≥ 2.5 to <5.0 | 11 | 1.2% | 18 | 2.0% | 31 | 3.5% | 580 | 64.8% | 255 | 28.5% | 895 |  |
|  | ≥ 5.0 to <7.5 | 2 | 0.5% | 9 | 2.1% | 35 | 8.1% | 272 | 62.8% | 115 | 26.6% | 433 |  |
|  | ≥ 7.5 | 9 | 1.3% | 21 | 3.0% | 29 | 4.2% | 548 | 78.5% | 91 | 13.0% | 698 |  |
| Mean of transportation to the health facility | | | |  |  |  |  |  |  |  |  | 3778 | <0.001 |
|  | Walking | 30 | 1.6% | 53 | 2.8% | 79 | 4.2% | 1111 | 58.5% | 627 | 33.0% | 1900 |  |
|  | Taxi mini bus | 6 | 0.5% | 17 | 1.5% | 57 | 5.1% | 636 | 56.4% | 411 | 36.5% | 1127 |  |
|  | Private car | 3 | 0.5% | 5 | 0.9% | 25 | 4.5% | 371 | 66.6% | 153 | 27.5% | 557 |  |
|  | Ambulance | 0 | 0.0% | 1 | 0.6% | 6 | 3.7% | 76 | 47.2% | 78 | 48.4% | 161 |  |
|  | Motorcycle/bicycle | 1 | 3.0% | 0 | 0.0% | 2 | 6.1% | 19 | 57.6% | 11 | 33.3% | 33 |  |
| Time taken from home to the health facility | | | |  |  |  |  |  |  |  |  | 3702 | 0.022 |
|  | < 30 minutes | 17 | 1.0% | 25 | 1.5% | 67 | 4.1% | 912 | 55.8% | 612 | 37.5% | 1633 |  |
|  | ≥ 30 min to <1 hour | 15 | 1.0% | 36 | 2.4% | 78 | 5.2% | 878 | 58.7% | 488 | 32.6% | 1495 |  |
|  | ≥ 1 hour to < 3 hour | 7 | 1.3% | 14 | 2.6% | 21 | 3.9% | 336 | 62.6% | 159 | 29.6% | 537 |  |
|  | ≥3 hours | 0 | 0.0% | 0 | 0.0% | 1 | 2.7% | 27 | 73.0% | 9 | 24.3% | 37 |  |
| Type of Health facility | |  |  |  |  |  |  |  |  |  |  | 3778 | <0.001 |
|  | Type II health centre | 29 | 1.3% | 53 | 2.3% | 90 | 4.0% | 1268 | 55.9% | 828 | 36.5% | 2268 |  |
|  | Type I health centre | 7 | 1.1% | 19 | 3.1% | 45 | 7.2% | 359 | 57.8% | 191 | 30.8% | 621 |  |
|  | Hospital | 4 | 0.4% | 4 | 0.4% | 34 | 3.8% | 586 | 65.9% | 261 | 29.4% | 889 |  |
| Referred to hospital | |  |  |  |  |  |  |  |  |  |  | 3778 | <0.001 |
|  | Yes | 1 | 0.3% | 1 | 0.3% | 12 | 3.9% | 144 | 46.6% | 151 | 48.9% | 309 |  |
|  | No | 39 | 1.1% | 75 | 2.2% | 157 | 4.5% | 2069 | 59.6% | 1129 | 32.5% | 3469 |  |
| Duration of labour | |  |  |  |  |  |  |  |  |  |  | 3400 | <0.001 |
|  | <12h | 12 | 1.8% | 23 | 3.4% | 15 | 2.2% | 369 | 54.1% | 263 | 38.6% | 682 |  |
|  | ≥ 12 to < 24h | 11 | 1.4% | 25 | 3.3% | 29 | 3.8% | 469 | 61.4% | 230 | 30.1% | 764 |  |
|  | ≥ 24h | 10 | 0.5% | 24 | 1.2% | 106 | 5.4% | 1164 | 59.6% | 650 | 33.3% | 1954 |  |
| Type of childbirth | |  |  |  |  |  |  |  |  |  |  | 3778 | 0.419 |
|  | Spontaneous vaginal | 40 | 1.1% | 76 | 2.1% | 161 | 4.4% | 2126 | 58.7% | 1216 | 33.6% | 3619 |  |
|  | Ventouse | 0 | 0.0% | 0 | 0.0% | 1 | 5.3% | 9 | 47.4% | 9 | 47.4% | 19 |  |
|  | Caesarean section | 0 | 0.0% | 0 | 0.0% | 7 | 5.0% | 78 | 55.7% | 55 | 39.3% | 140 |  |
|  |  |  |  |  |  |  |  |  |  |  |  |  |  |
| Presence of companion throughout the childbirth | | | | | |  |  |  |  |  |  | 3778 | <0.001 |
|  | Yes | 15 | 0.7% | 35 | 1.7% | 102 | 4.9% | 1188 | 56.7% | 755 | 36.0% | 2095 |  |
|  | No | 25 | 1.5% | 41 | 2.4% | 67 | 4.0% | 1025 | 60.9% | 525 | 31.2% | 1683 |  |
| Type of companion | |  |  |  |  |  |  |  |  |  |  | 2095 | <0.001 |
|  | Mother-in-law | 5 | 0.5% | 22 | 2.3% | 58 | 6.0% | 528 | 54.4% | 358 | 36.9% | 971 |  |
|  | Mother | 5 | 1.5% | 5 | 1.5% | 17 | 5.0% | 186 | 54.5% | 128 | 37.5% | 341 |  |
|  | Husband/partner | 0 | 0.0% | 0 | 0.0% | 2 | 1.9% | 92 | 86.8% | 12 | 11.3% | 106 |  |
|  | Friend/neighbour | 0 | 0.0% | 3 | 2.5% | 9 | 7.6% | 76 | 63.9% | 31 | 26.1% | 119 |  |
|  | Others | 5 | 0.9% | 5 | 0.9% | 16 | 2.9% | 306 | 54.8% | 226 | 40.5% | 558 |  |
| Outcome | |  |  |  |  |  |  |  |  |  |  | 3778 | 0.680 |
|  | Livebirth | 38 | 1.0% | 73 | 2.0% | 165 | 4.5% | 2163 | 58.6% | 1252 | 33.9% | 3691 |  |
|  | Stillbirth | 2 | 2.3% | 3 | 3.4% | 4 | 4.6% | 50 | 57.5% | 28 | 32.2% | 87 |  |
